# CLIMATE CHANGE FOR OLDER INDIVIDUALS EXPERIENCING HOMELESSNESS: CONSIDERATIONS FOR DISASTER EVENTS

**DOI:** 10.1093/geroni/igz038.1991

**Published:** 2019-11-08

**Authors:** Allison Gibson

**Affiliations:** University of Kentucky, Lexington, Kentucky, United States

## Abstract

There is a growing field of evidence that individuals experiencing homelessness are disproportionately impacted by climate change due to factors like exposure to the elements, lack of resources and services, as well as disenfranchisement and stigma; all while experiencing greater occurrences of environmental injustice. Given that there are distinct needs for older individuals experiencing homelessness when affected by disasters, this study will report salient themes identified from qualitative interviews with five residential shelters on considerations they incorporate when disaster planning for their older residents. Thematic analysis revealed older age and homelessness have serious implications for planning, responding and in the recovery of disasters. Challenges to finding accessible transitional and permanent housing, limitations to workforce re-entry, increasing income inequality between classes, limitations to mental health services and policies, and cultural justifications for criminalizing poverty and homelessness contribute to our collective understanding of disaster vulnerability when older adults experience homelessness.

